# Anti-Inflammatory and Skin-Lightening Activities of Exosomes Derived from *Latilactobacillus sakei* BK-5 Isolated from *Aster koraiensis* Flowers

**DOI:** 10.4014/jmb.2511.11022

**Published:** 2025-12-15

**Authors:** Chae-Yeon Lee, Byeong-Min Choi, Da Som Kim, Hyeri Choi, Hyehyun Hong, Won-Jae Chi, Seung-Young Kim

**Affiliations:** 1 Department of Pharmaceutical Engineering & Biotechnology, Sunmoon University, Chungnam 31460, Republic of Korea; 2 Species Diversity Research Division, National Institute of Biological Resources, Incheon 22689, Republic of Korea

**Keywords:** Anti-inflammation, *Aster koraiensis*; *Latilactobacillus sakei* BK-5, MAPK, Melanogenesis

## Abstract

Exosomes are nanosized vesicles released from multivesicular bodies into the extracellular space. Lactic acid bacteria-derived exosomes have emerged as promising next-generation bioactive agents with beneficial effects on skin health and immune regulation. In this study, we aimed to investigate the anti-inflammatory and skin-lightening activities of exosomes derived from *Latilactobacillus sakei* isolated from the flowers of *Aster koraiensis* (BK-5 exosomes). Experiments were performed using lipopolysaccharide-stimulated RAW 264.7 macrophages and α-melanocyte-stimulating hormone-induced B16F10 melanoma cells. BK-5 exosomes exhibited no cytotoxicity in either cell line and effectively suppressed the production of proinflammatory cytokines, including interleukin (IL)-6, IL-1β, tumor necrosis factor-α, and prostaglandin E_2_, as well as the expression of inducible nitric oxide synthase and cyclooxygenase-2. Moreover, BK-5 exosomes reduced the expression of melanogenesis-related proteins, including tyrosinase, tyrosinase-related protein (TRP)-1, TRP-2, and microphthalmia-associated transcription factor in a dose-dependent manner. Furthermore, BK-5 exosomes inhibited the phosphorylation of nuclear factor kappa-light-chain-enhancer of activated B cells and inhibitor of κB alpha, as well as the phosphorylation of mitogen-activated protein kinase pathway components, including extracellular signal-regulated kinase, c-Jun N-terminal kinase, and p38. Collectively, these findings confirm that BK-5 exosomes effectively attenuate inflammatory responses in RAW 264.7 macrophages through suppression of multiple signaling pathways and inhibit melanin biosynthesis in B16F10 cells, thereby demonstrating their potential as multifunctional bioactive materials applicable to pharmaceutical and cosmetic fields.

## Introduction

Inflammation is an innate defense mechanism of the host against harmful stimuli such as pathogens, tissue injury, and toxic substances. It facilitates the infiltration of leukocytes and plasma components into the damaged site, thereby contributing to pathogen elimination and tissue repair. Acute inflammation is accompanied by classical symptoms, including redness, swelling, heat, and pain, and serves a physiologically protective role [1]. However, when this response becomes excessive or persists over an extended period, it can lead to tissue damage and pathological alterations, ultimately contributing to the development of various chronic diseases such as cancer, cardiovascular disorders, and autoimmune diseases [2]. Therefore, elucidating the molecular mechanisms underlying inflammatory responses and establishing strategies to regulate them are critical to promoting human health and preventing disease.

Macrophages play a central role in the initiation of inflammation. Upon stimulation by agents such as lipopolysaccharide (LPS), the expression of inducible nitric oxide synthase (iNOS) and cyclooxygenase (COX)-2 is induced, leading to the production of inflammatory mediators including nitric oxide (NO) and prostaglandin E_2_ (PGE_2_) [3]. Furthermore, the secretion of key proinflammatory cytokines such as interleukin (IL)-6, IL-1β, and tumor necrosis factor-α (TNF-α) amplifies the inflammatory response and contributes to its chronic progression. These processes are intricately regulated by intracellular signaling pathways, including the nuclear factor kappa-light-chain-enhancer of activated B cells (NF-κB) and mitogen-activated protein kinase (MAPK) cascades, which play pivotal roles in understanding the pathophysiology of inflammatory diseases and identifying novel therapeutic targets [4-7].

Melanin is a pigment that protects the skin from ultraviolet (UV) radiation; however, its excessive production can cause cutaneous disorders, such as melasma, freckles, and hyperpigmentation. The process of melanogenesis is primarily regulated by the binding of alpha-melanocyte-stimulating hormone (α-MSH) to the melanocortin 1 receptor, which activates the cyclic adenosine monophosphate (cAMP)–protein kinase A–cAMP response element-binding protein signaling axis. This activation enhances the expression of tyrosinase and tyrosinase-related proteins (TRP)-1 and TRP-2 via microphthalmia-associated transcription factor (MITF) [8, 9]. Additionally, the MAPK and phosphatidylinositol 3-kinase/protein kinase B (AKT) pathways further modulate the stability and transcriptional activity of MITF [10, 11]. The discovery of effective agents capable of regulating melanin synthesis has become a key research focus for the development of skin-lightening products and the improvement of pigmentation disorders.

Exosomes are nanosized extracellular vesicles (30–200 nm) that originate from multivesicular bodies within cells and are secreted into the extracellular environment. They contain various bioactive molecules, including proteins, lipids, messenger RNA (mRNA), and microRNA (miRNA), and participate in a wide range of physiological and pathological processes such as intercellular communication, immune regulation, and tissue regeneration [12, 13]. Due to their stability in body fluids, exosomes serve not only as promising biomarkers for disease diagnosis but also as potent drug delivery vehicles capable of transferring exogenous substances into recipient cells [14, 15]. Although various methods, including ultracentrifugation and precipitation techniques, have been developed for exosome isolation and purification, these approaches often face limitations in terms of yield, purity, and structural integrity. Tangential flow filtration (TFF), which allows for rapid processing of large sample volumes while minimizing structural damage and effectively removing impurities, was therefore employed as the purification method in this study [16-18].

Recent reports have indicated that plant-derived lactic acid bacteria (LAB) exhibit multiple physiological benefits beyond gut microbiota modulation, including reinforcement of the skin barrier, antioxidative activity, and immune regulation [19-21]. Notably, exosomes secreted by these bacteria contain bioactive molecules-proteins, nucleic acids, and metabolites—that reflect the physiological characteristics of the host, thereby representing a novel class of functional materials that transcend the traditional limitations of probiotics [22, 23]. Unlike conventional probiotics, which mainly act within the intestinal environment, exosomes possess the unique ability to mediate direct intercellular communication and deliver bioactive substances to specific target tissues [24, 25]. Based on these distinctive properties, recent studies have highlighted the potential application of LAB-derived exosomes as next-generation biofunctional agents for skin health and immune modulation [26]. Furthermore, several studies have reported the significant potential of plant-derived LAB exosomes for use in developing multifunctional skin-care materials with anti-inflammatory, antioxidant, skin-lightening, and tissue-regenerative properties [27, 28]. Accordingly, BK-5 exosomes derived from *Latilactobacillus sakei* isolated from *Aster koraiensis* flowers were employed in this study to further validate their potential as multifunctional bioactive agents with anti-inflammatory and skin-lightening properties.

In this study, we investigated the anti-inflammatory and skin-lightening activities of exosomes derived from *L. sakei* (BK-5 exosomes), isolated from the flowers of *A. koraiensis*, a native Korean plant. BK-5 exosomes effectively suppressed LPS-induced NO production in RAW 264.7 macrophages by downregulating iNOS and COX-2 and inhibiting the NF-κB and MAPK signaling pathways [29, 30]. Furthermore, α-MSH-induced melanogenesis in B16F10 melanoma cells was attenuated by BK-5 exosomes through the suppression of MITF expression and subsequent downregulation of key melanogenic enzymes, including tyrosinase, TRP-1, and TRP-2 [31, 32]. Collectively, these findings suggest that plant-derived LAB exosomes possess dual regulatory activities against inflammation and melanogenesis, highlighting their potential as next-generation biofunctional materials for skin applications.

## Materials and Methods

### Culture of *Latilactobacillus sakei* BK-5

The *Latilactobacillus sakei* BK-5 strain was isolated from *Aster koraiensis* flowers collected on Seo-gu district of Incheon, Republic of Korea in 2019, and was provided by the National Institute of Biological Resources (NIBR, Republic of Korea). *L. sakei* BK-5 was cultured under aerobic conditions at 28°C using de Man, Rogosa, and Sharpe (MRS) medium, which supports its optimal growth. Prior to experiments, the strain was cultured in MRS broth at 28°C with shaking at 200 rpm for 18 h.

### Exosome Isolation

After cultivation, bacterial cells were removed by centrifugation at 4,000 ×*g* for 15 min, and the resulting supernatant was filtered through a 0.22 μm pore-size vacuum filter (SPL, Republic of Korea). The exosomes were then purified using an ÄKTA flux S system (Cytiva, UK) equipped with a TFF module [33]. The TFF system employed a hollow fiber cartridge with an internal fiber diameter of 0.5 mm, a flow path length of 110 cm, and a nominal molecular weight cutoff of 100,000 Da.

### Characterization of BK-5 Derived Exosomes

The physicochemical characteristics of the exosomes were analyzed using nanoparticle tracking analysis (NTA) and transmission electron microscopy (TEM) [34]. NTA was performed with a ZetaView PMX 110 instrument (Particle Metrix, Germany) and ZetaView software (version 8.05.16 SP3). For TEM observation, samples were placed onto formvar-coated copper grids, stained with 2% uranyl acetate for 20 s, blotted to remove excess stain, and imaged using an Alos L120C transmission electron microscope (FEI, USA).

### Materials and Cell Culture

α-MSH, LPS, Griess reagent, and L-3,4-dihydroxyphenylalanine (L-DOPA) were purchased from Sigma-Aldrich (USA). B16F10 melanoma cells and RAW 264.7 macrophages were obtained from the American Type Culture Collection (USA). Cells were cultured in Dulbecco’s Modified Eagle’s Medium (Welgene, Republic of Korea) supplemented with 10% fetal bovine serum (Welgene), 100 μg/ml penicillin, and 100 μg/ml streptomycin. RAW 264.7 and B16F10 cells were subcultured every 2 and 3 days, respectively, and maintained in a humidified incubator at 37°C with 5% CO_2_.

### Measurement of 2,2'-Azino-bis(3-ethylbenzothiazoline-6-sulfonic acid) (ABTS) Radical Scavenging Activity

The ABTS radical scavenging activity was determined with slight modifications of the method described by Van den Berg *et al*. [35, 36]. Equal volumes of 7.4 mM ABTS and 2.6 mM potassium persulfate (K_2_S_2_O_8_) solutions were mixed and incubated in the dark for at least 24 h to generate the ABTS•^+^ (radical cation). The resulting solution was diluted with distilled water before use. To evaluate radical scavenging activity, 20 μl of BK-5 exosome samples (1.69 × 10^7^, 3.38 × 10^7^, and 6.75 × 10^7^ particles/ml) were mixed with 180 μl of the ABTS radical solution and incubated for 10 min in the dark. The absorbance (A) of each sample was then measured and compared with that of the control. The radical scavenging activity was calculated using the following equation:

*ABTS radical scavenging activity (%)* = [1 – (*A_sample_/A_control_*) × 100

### Cytotoxicity Measurement

To evaluate the effect of *L. sakei*–derived exosomes (BK-5 exosomes) on the viability of LPS-stimulated RAW 264.7 cells and α-MSH-stimulated B16F10 melanoma cells, a 3-(4,5-dimethylthiazol-2-yl)-2,5-diphenyltetrazolium bromide (MTT) assay was performed. RAW 264.7 cells were seeded at a density of 8 × 10^4^ cells/well in 24-well plates and pre-incubated for 24 h at 37°C in a 5% CO_2_ incubator. Subsequently, the cells were co-treated with 1 μg/ml LPS and BK-5 exosomes (1.69 × 10^7^, 3.38 × 10^7^, and 6.75 × 10^7^ particles/ml) for 24 h. Similarly, B16F10 cells were seeded at 1 × 10^4^ cells/well under the same conditions and pre-incubated for 24 h. The cells were then co-treated with 200 nM α-MSH and BK-5 exosomes (8.45 × 10^6^, 1.69 × 10^7^, and 3.38 × 10^7^ particles/ml) for 72 h. Following incubation, MTT (Sigma-Aldrich) was added to each well and incubated for 3 h at 37°C in a 5% CO_2_ atmosphere to allow for the formation of insoluble formazan crystals [37]. The supernatant was removed, and dimethyl sulfoxide (Sigma-Aldrich) was added to dissolve the formazan. The resulting solution was transferred to a 96-well plate, and absorbance was measured at 570 nm using a microplate reader (Thermo Fisher Scientific, USA).

### Measurement of NO Production Inhibition

To evaluate the inhibitory effect of BK-5 exosomes on NO production, RAW 264.7 cells were seeded at a density of 8 × 10^4^ cells/well in 24-well plates and incubated for 24 h at 37°C in a 5% CO_2_ atmosphere. The cells were then co-treated with LPS (1 μg/ml) and BK-5 exosomes (1.69 × 10^7^, 3.38 × 10^7^, and 6.75 × 10^7^ particles/ml) for 24 h. Following treatment, 100 μl of culture supernatant was mixed with an equal volume of Griess reagent [1% (w/v) sulfanilamide and 0.1% (w/v) naphthylethylenediamine in 2.5% (v/v) phosphoric acid] and incubated for 10 min in the dark [38]. The absorbance was measured at 540 nm using a microplate reader to quantify NO levels.

### Measurement of PGE_2_ Production Inhibition

RAW 264.7 cells were seeded at 8 × 10^4^ cells/well in 24-well plates and pre-incubated for 24 h. The cells were then treated with LPS (1 μg/ml) and BK-5 exosomes (1.69 × 10^7^, 3.38 × 10^7^, and 6.75 × 10^7^ particles/ml) for 24 h. The culture media were collected and centrifuged at 12,000 ×*g* for 3 min, and the supernatants were used for analysis. The concentration of PGE_2_ was determined using a Mouse PGE_2_ ELISA Kit (R&D Systems Inc., USA).

### Measurement of TNF-α, IL-6, and IL-1β Production Inhibition

RAW 264.7 cells were seeded at a density of 8 × 10^4^ cells/well in 24-well plates and incubated for 24 h. The cells were then treated with LPS (1 μg/ml) and BK-5 exosomes (1.69 × 10^7^, 3.38 × 10^7^, and 6.75 × 10^7^ particles/ml) for 24 h. After treatment, the culture supernatants were collected by centrifugation at 12,000 ×*g* for 3 min. The concentrations of TNF-α, IL-6, and IL-1β were measured using the Mouse TNF-α ELISA Kit (Invitrogen, USA), Mouse IL-6 ELISA Kit (BD Biosciences, USA), and Mouse IL-1β ELISA Kit (R&D Systems Inc., USA), respectively.

### Measurement of Melanin Content

B16F10 melanoma cells were seeded at a density of 4 × 10^4^ cells/well in 6-well plates and incubated for 24 h. The cells were then co-treated with 200 nM α-MSH and BK-5 exosomes (8.45 × 10^6^, 1.69 × 10^7^, and 3.38 × 10^7^ particles/ml) for 72 h. After incubation, the cells were detached using trypsin and lysed for 60 min in radioimmunoprecipitation assay (RIPA) buffer (Biosesang, Republic of Korea) containing 1 mM sodium orthovanadate (Na_3_VO_4_), 1 mM phenylmethylsulfonyl fluoride (PMSF), and 1% protease inhibitor. The lysates were centrifuged at 13,000 ×*g* for 30 min at 4°C, and the supernatants were discarded. The resulting pellets were dissolved in 500 μl of 1 N NaOH and heated at 90°C for 1 h. The final solutions were transferred to a 96-well plate, and the absorbance was measured at 405 nm using a microplate reader.

### Measurement of Tyrosinase Activity

B16F10 melanoma cells were seeded at a density of 4 × 10^4^ cells/well in 6-well plates and pre-incubated for 24 h at 37°C in a 5% CO_2_ incubator. The cells were then treated with 200 nM α-MSH and BK-5 exosomes (8.45 × 10^6^, 1.69 × 10^7^, and 3.38 × 10^7^ particles/ml) and incubated for 72 h. After incubation, the cells were detached using trypsin and lysed in RIPA buffer containing 1 mM Na_3_VO_4_, 1 mM PMSF, and 1% protease inhibitor for 60 min. The lysates were centrifuged at 13,000 ×*g* for 30 min at 4°C to obtain the supernatant, and the protein concentration was determined using the Pierce BCA Protein Assay Kit (Thermo Fisher Scientific). Subsequently, 20 μl of the quantified protein samples were mixed with 80 μl of 2 mg/ml L-DOPA solution and incubated for 2 h in the dark. The absorbance was then measured at 490 nm using a microplate reader [39].

### Western Blotting

RAW 264.7 cells (5 × 10^5^ cells/well) were seeded in 6-well plates and incubated for 24 h at 37°C in a 5% CO_2_ atmosphere, followed by co-treatment with LPS (1 μg/ml) and BK-5 exosomes (1.69 × 10^7^, 3.38 × 10^7^, and 6.75 × 10^7^ particles/ml) for 24 h. Similarly, B16F10 melanoma cells (4 × 10^4^ cells/well) were seeded in 6-well plates and pre-incubated for 48 h under the same conditions before co-treatment with 200 nM α-MSH and BK-5 exosomes (8.45×106, 1.69 × 10^7^, and 3.38 × 10^7^ particles/ml) for 24 h. After incubation, the cells were collected by centrifugation and lysed in RIPA buffer (Biosesang) containing 1 mM Na_3_VO_4_, 1 mM PMSF, and 1% protease inhibitor for 60 min. The lysates were centrifuged at 13,000 ×*g* for 30 min at 4°C, and the protein concentrations of the supernatants were determined using the Pierce BCA Protein Assay Kit (Thermo Fisher Scientific). Equal amounts of protein (20 μg) were separated by 10% sodium dodecyl sulfate–polyacrylamide gel electrophoresis and transferred to polyvinylidene difluoride membranes (Millipore, USA). The membranes were blocked with 5%skim milk in 1× Tris-buffered saline containing 0.1% Tween 20 (TBST) for 2 h at room temperature and washed three times for 10 min each with TBST. The membranes were then incubated overnight at 4°C with primary antibodies against iNOS (1:1,000; Cell Signaling Technology, USA), COX-2 (1:1,000; Cell Signaling), β-actin (1:1,000; Cell Signaling), phospho-p44/42 MAPK (Erk1/2; Thr202/Tyr204; 1:500; Cell Signaling), phospho-SAPK/c-Jun N-terminal kinase (JNK; 1:500; Cell Signaling), phospho-p38 MAPK (Thr180/Tyr182; 1:500; Cell Signaling), total p44/42 MAPK (Erk1/2; 1:500; Cell Signaling), SAPK/JNK (1:500; Cell Signaling), p38 MAPK (1:500; Cell Signaling), phospho-NF-κB (1:1,000; Cell Signaling), inhibitor of κB alpha (IκB-α; 1:1,000; Cell Signaling), TRP-1 (1:5,000; Santa Cruz Biotechnology, USA), TRP-2 (1:500; Santa Cruz), tyrosinase (1:500; Santa Cruz), and MITF (1:500; Cell Signaling). After washing three times with TBST, the membranes were incubated for 2 h at room temperature with horseradish peroxidase-conjugated anti-mouse immunoglobulin G (IgG) or anti-rabbit IgG secondary antibodies (1:10,000; Cell Signaling). The membranes were washed three times with TBST and visualized using an ECL Detection Kit (Bio-Rad, USA). Protein bands were detected using an LAS 4000 mini imaging system (Fujifilm, Japan) and quantified using ImageJ software version 1.52v (NIH, USA) [40].

### Statistical Analysis

All experiments were conducted in triplicate, and the results were expressed as mean ± standard deviation. Statistical significance among treatment groups was evaluated using analysis of variance, followed by Student’s t-test for multiple comparisons. Significance thresholds of *p < 0.05; **p < 0.01; and ***p < 0.001 were applied.

## Results

### Exosome Analysis

**Fig. 1:**
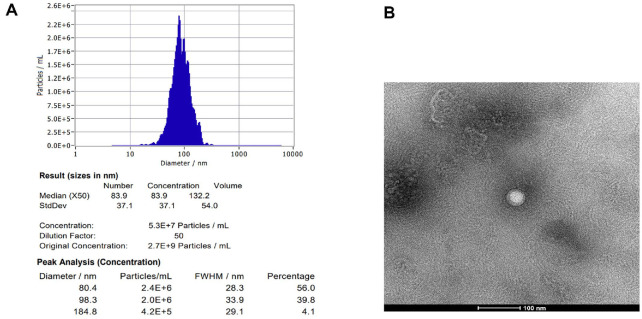
Characterization of *Latilactobacillus sakei* BK-5 derived exosomes (BK-5 exosomes). (**A**) Nanoparticle tracking analysis (NTA) of BK-5 exosomes. (**B**) Transmission Electron Microscopy image of BK-5 exosomes.

The analysis revealed that the concentration of BK-5 exosome samples was 2.7 × 10^9^ particles/ml, with most particles distributed within the 50–200 nm range (Fig. 1A). TEM further confirmed the presence of spherical vesicles with a well-defined membrane structure (Fig. 1B).

### Evaluation of ABTS Radical Scavenging Activity

**Fig. 2:**
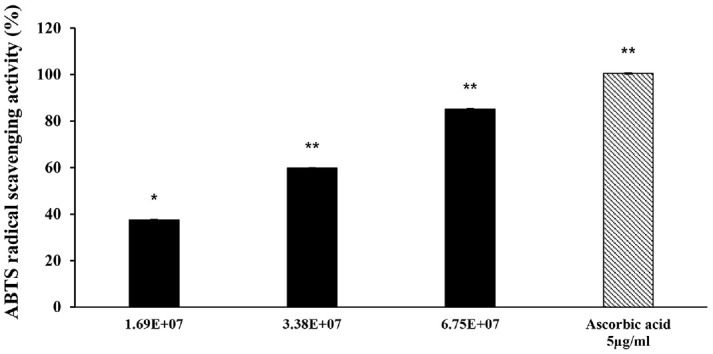
ABTS radical scavenging activity of BK-5 exosomes. Results are expressed as percentages compared to the respective values obtained for the control. ABTS, 2,2'-Azino-bis(3-ethylbenzothiazoline-6-sulfonic acid); BK-5 exosomes, *Latilactobacillus sakei*–derived exosomes.

Evaluation of the antioxidant activity of BK-5 exosomes using the ABTS radical assay, with absorbance measured at 734 nm, showed a concentration-dependent increase in scavenging activity. BK-5 exosomes exhibited radical scavenging activities of 37.54%, 59.74%, and 85.18% at concentrations of 1.69 × 10^7^, 3.38 × 10^7^, and 6.75 × 10^7^ particles/ml, respectively (Fig. 2). These findings indicate that BK-5 exosomes consistently exert antioxidant effects across all tested concentrations, with efficacy expressed relative to particle number.

### Cell Viability

**Fig. 3:**
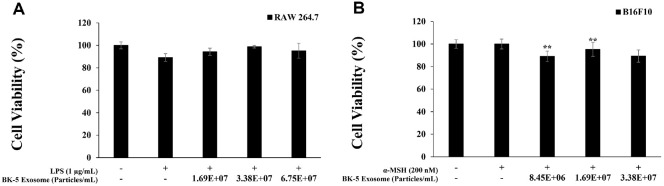
Cell viability of BK-5 exosomes on lipopolysaccharide (LPS)-stimulated RAW 264.7 cells and alphamelanocyte- stimulating hormone (α-MSH)-induced B16F10 melanoma cells. (**A**) Cell viability of RAW 264.7 macrophage cells. (**B**) Cell viability of B16F10 melanoma cells. The cytotoxicity of RAW 264.7 cells was determined using the 3-(4,5-dimethylthiazol-2-yl)-2,5-diphenyl-2H-tetrazolium bromide (MTT) assay for LPS (1 μg/ml)-stimulated cells in the presence of BK-5 exosomes (1.69 × 10^7^, 3.38 × 10^7^, 6.75 × 10^7^ particles/ml). The cytotoxicity of B16F10 cells was determined using the MTT assay for α-MSH (200 nM)-stimulated cells in the presence of BK-5 exosomes (8.45 × 10^6^, 1.69 × 10^7^, 3.38 × 10^7^ particles/ml). Results are expressed as percentages relative to the control. **p < 0.01. BK-5 exosomes, *Latilactobacillus sakei*–derived exosomes.

The viability of RAW 264.7 macrophages treated with LPS (1 μg/ml) and BK-5 exosomes (1.69 × 10^7^, 3.38 × 10^7^, and 6.75 × 10^7^ particles/ml), as well as that of B16F10 melanoma cells treated with α-MSH (200 nM) and BK-5 exosomes (8.45 × 10^6^, 1.69 × 10^7^, and 3.38 × 10^7^ particles/ml), were assessed using MTT assay. Both cell lines maintained viability above 80% at all tested concentrations (Fig. 3A and 3B), indicating that BK-5 exosomes did not induce cytotoxicity within this concentration range [41].

### Inhibition of NO Expression

**Fig. 4:**
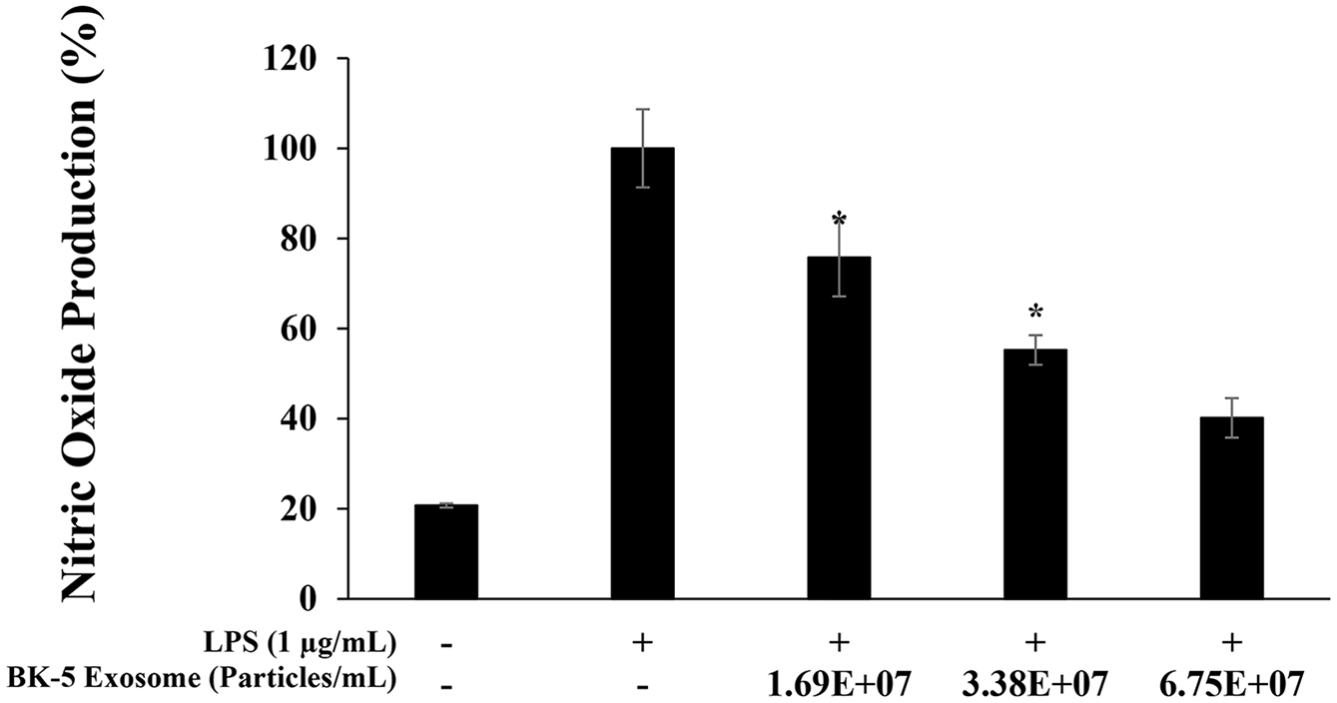
Nitric oxide production inhibition by BK-5 exosomes in LPS-stimulated RAW 264.7 cells. The production of nitric oxide in 1 μg/ml LPS-stimulated RAW 264.7 cells in the presence of BK-5 exosomes (1.69 × 10^7^, 3.38 × 10^7^, 6.75 × 10^7^ particles/ml). The results are expressed as percentages compared with the respective values obtained for the control. Data represent the means ± standard deviation with three independent experiments. *p < 0.05. BK-5 exosomes, *Latilactobacillus sakei*–derived exosomes; LPS, lipopolysaccharide.

Co-treatment of RAW 264.7 macrophages with LPS and BK-5 exosomes resulted in a dose-dependent suppression of NO production. At the highest concentration (6.75 × 10^7^ particles/ml), NO levels were markedly reduced, approaching those observed in the untreated control group (Fig. 4).

### Inhibition of PGE_2_ Expression

**Fig. 5:**
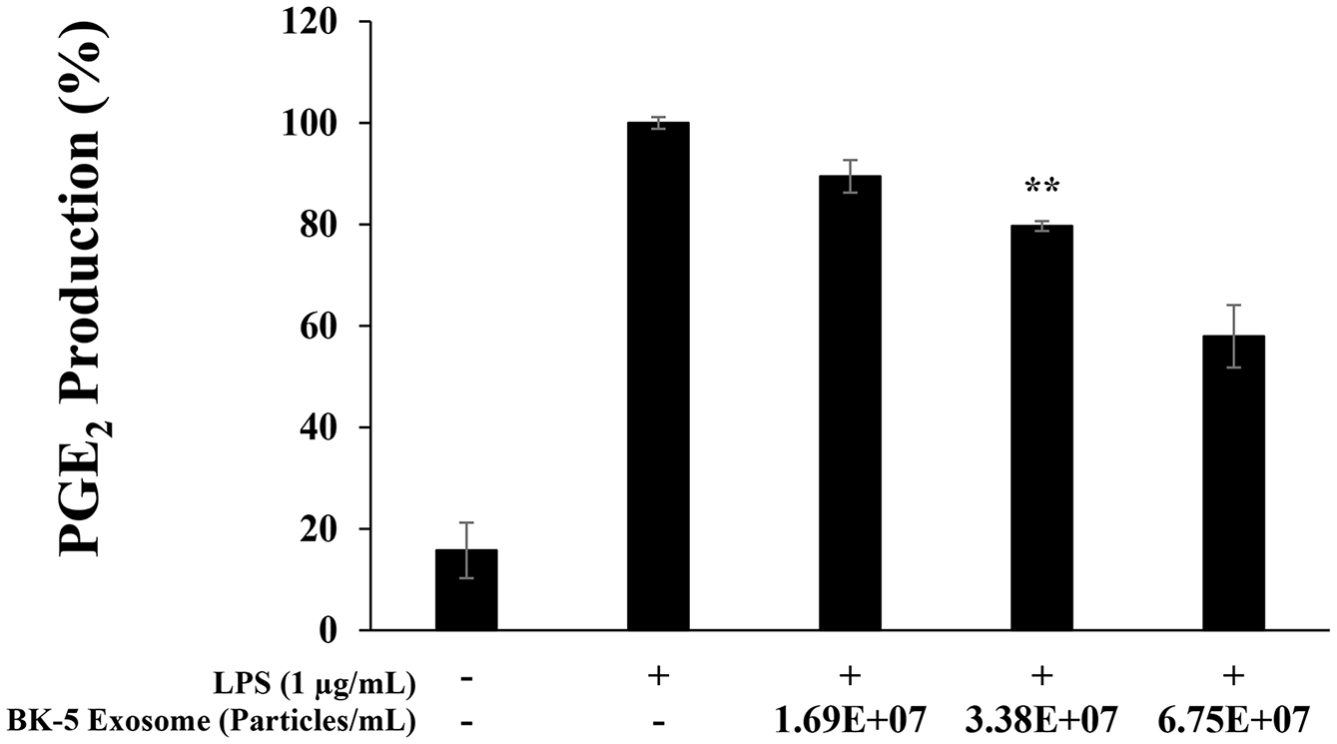
Inhibitory effects of BK-5 exosomes on prostaglandin E_2_ (PGE_2_) production in LPS-stimulated RAW 264.7 cells. PGE_2_ production in 1 μg/ml LPS-stimulated RAW 264.7 cells in the presence of BK-5 exosomes (1.69 × 10^7^, 3.38 × 10^7^, 6.75 × 10^7^ particles/ml). Results are expressed as percentages compared with the respective values obtained for the control. Data represent the means ± standard deviation with three independent experiments. **p < 0.01. BK-5 exosomes, *Latilactobacillus sakei*–derived exosomes; LPS, lipopolysaccharide.

To evaluate the inhibitory effect of BK-5 exosomes on PGE_2_ production in LPS-stimulated RAW 264.7 macrophages, cells were co-treated with LPS (1 μg/ml) and BK-5 exosomes at concentrations of 1.69 × 10^7^, 3.38 × 10^7^ and 6.75 × 10^7^ particles/ml. BK-5 exosomes markedly reduced PGE_2_ production in a dose-dependent manner, showing a significant 42% decrease at the highest concentration (6.75 × 10^7^ particles/ml) compared with the LPS-treated control group (Fig. 5).

### Inhibition of Proinflammatory Cytokine Expression

**Fig. 6:**
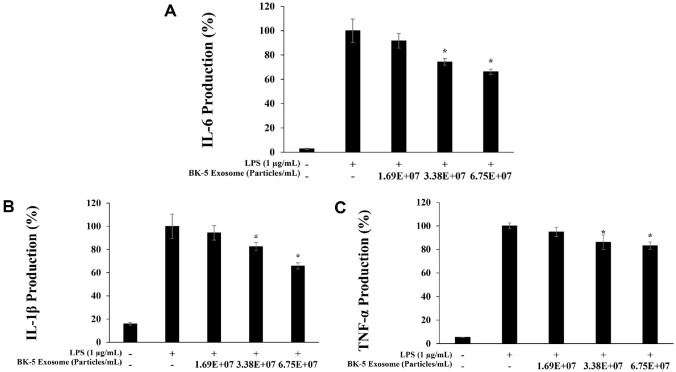
Inhibition of proinflammatory cytokines by BK-5 exosomes in LPS-stimulated RAW 264.7 cells. Production of (**A**) interleukin (IL)-6, (**B**) IL-1β, and (**C**) tumor necrosis factor alpha (TNF-α) in LPS (1 μg/ml)-stimulated RAW 264.7 cells in the presence of BK-5 exosomes (1.69 × 10^7^, 3.38 × 10^7^, 6.75 × 10^7^ particles/ml). Data represent the means ± standard deviation with three independent experiments. *p < 0.05. BK-5 exosomes, *Latilactobacillus sakei*–derived exosomes; LPS, lipopolysaccharide.

Macrophages stimulated by LPS secrete various proinflammatory cytokines that amplify the inflammatory response, which is closely linked to NO and PGE_2_ production [42]. BK-5 exosomes suppressed the expression of proinflammatory cytokines in a dose-dependent manner. IL-6 and IL-1β levels were significantly reduced in a concentration-dependent manner. In contrast, TNF-α exhibited only a slight reduction trend, with a relatively limited inhibitory effect (Fig. 6A-6C).

### Inhibition of Melanin Production

**Fig. 7:**
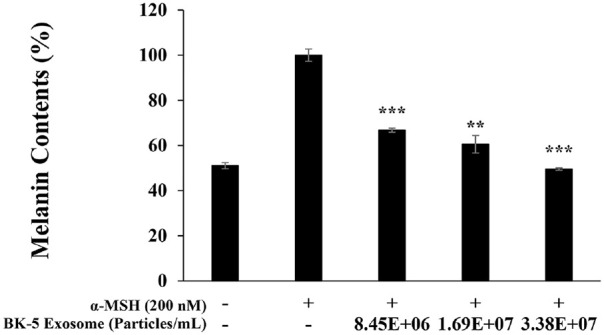
Effect of BK-5 exosomes on melanin synthesis in B16F10 melanoma cells. The production of melanin was assayed in the cell pellets of α-MSH (200 nM)-stimulated cells for 72 h in the presence of BK-5 exosomes (8.45 × 10^6^, 1.69 × 10^7^, and 3.38 × 10^7^ particles/ml). Melanin levels were expressed as percentages normalized to the α-MSH–treated group, which was set to 100%. Data represent the means ± standard deviation with three separate experiments. **p < 0.01; ***p < 0.001. BK-5 exosomes, *Latilactobacillus sakei*–derived exosomes; α-MSH, alpha-melanocyte-stimulating hormone.

BK-5 exosomes induced a concentration-dependent decrease in melanin content, with the highest concentration (3.38 × 10^7^ particles/ml) reducing melanin levels to those observed in the untreated control group (Fig. 7). Thereafter, to determine whether BK-5 exosomes could serve as potential inhibitors of melanogenesis, the inhibitory effect on tyrosinase activity, a key rate limiting enzyme in the early stage of melanin synthesis, was investigated.

### Inhibition of Tyrosinase Activity

**Fig. 8:**
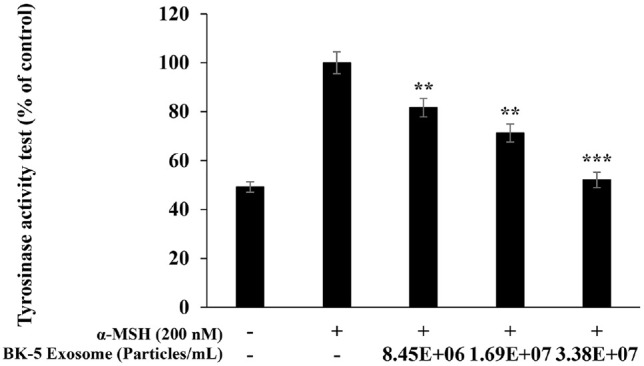
Effect of BK-5 exosomes on tyrosinase activity in B16F10 melanoma cells. The cells were stimulated with α-MSH (200 nM for 72 h in the presence of BK-5 exosomes (8.45 × 10^6^, 1.69 × 10^7^, and 3.38 × 10^7^ particles/ml). The effect of BK- 5 exosomes on tyrosinase activity was determined by measuring the absorbance at 490 nm, and all values were expressed as percentages normalized to the α-MSH–treated group, which was set to 100%. Data represent the mean ± standard deviation of three independent experiments. **p < 0.01; ***p < 0.001. BK-5 exosomes, *Latilactobacillus sakei*–derived exosomes; α-MSH, alpha-melanocyte-stimulating hormone.

Tyrosinase is a key enzyme in the melanin biosynthesis pathway that catalyzes the oxidation of L-tyrosine into L-DOPA and subsequently to DOPA-quinone [43]. Therefore, we assessed the effects of BK-5 exosomes on the inhibition of tyrosinase activity. Co-treatment of α-MSH–stimulated B16F10 melanoma cells with BK-5 exosomes (8.45 × 10^6^, 1.69 × 10^7^, and 3.38 × 10^7^ particles/ml) resulted in a concentration-dependent decrease in tyrosinase activity, with a statistically significant reduction observed at the highest concentration (3.38 × 10^7^ particles/ml)(Fig. 8).

### Inhibitory Effects on iNOS and COX-2 Expression

To determine whether the reduction in NO and PGE_2_ production was associated with the inhibition of iNOS and COX-2 expression, Western blot analysis was performed. The results showed that BK-5 exosome treatment decreased the protein expression levels of iNOS and COX-2 in a concentration-dependent manner, with a significant suppression observed at the highest concentration.[Fig F9]

### Inhibition Effects of MAPK Phosphorylation by BK-5 Exosomes

**Fig. 10:**
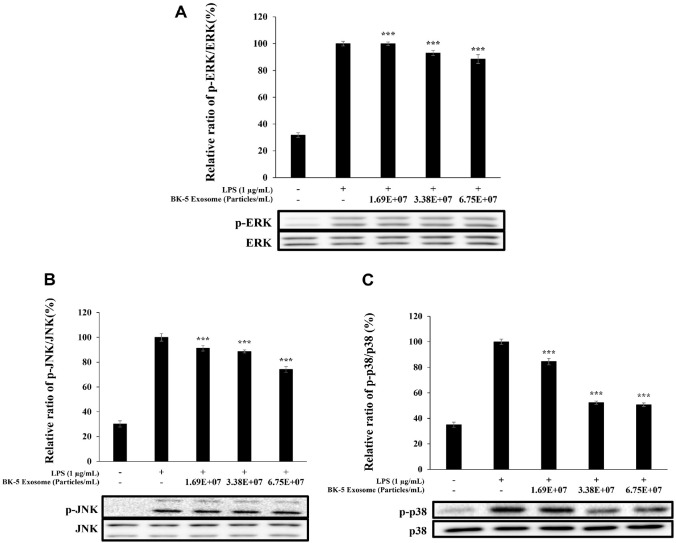
Effect of BK-5 exosomes on MAPK phosphorylation in RAW 264.7 cells. Cells were preincubated for 24 h and then treated with 1 μg/ml LPS and BK-5 exosomes (1.69 × 10^7^, 3.38 × 10^7^, 6.75 × 10^7^ particles/ml). (**A**) p-ERK/ERK, (**B**) p- JNK/JNK, (**C**) p-p38/p38. Protein levels were analyzed using western blotting. Data represent the means ± standard deviation with three independent experiments. ***p < 0.001. BK-5 exosomes, *Latilactobacillus sakei*–derived exosomes; LPS, lipopolysaccharide; MAPK, mitogen-activated protein kinase; ERK, extracellular signal-regulated kinase; p-ERK, phosphorylated ERK; JNK, c- Jun N-terminal kinase; p-JNK, phosphorylated JNK.

Treatment with BK-5 exosomes resulted in a gradual decrease in ERK phosphorylation with increasing concentrations of BK-5 exosomes, although the inhibition was relatively moderate. JNK phosphorylation exhibited a similar downward trend without pronounced suppression. In contrast, p38 phosphorylation was markedly reduced in a dose-dependent manner, with significant inhibition observed at the highest concentration (6.75 × 10^7^ particles/ml) (Fig. 10).

### Inhibition of the NF-κB Signaling Pathway

**Fig. 11:**
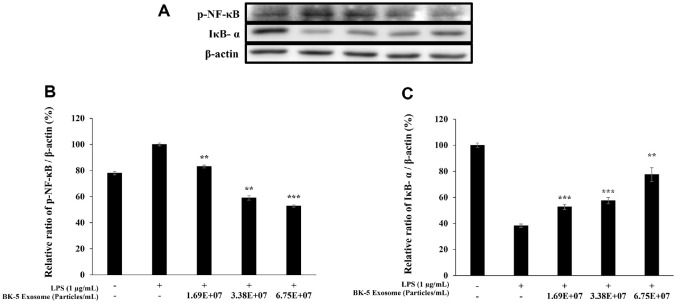
Effect of BK-5 exosomes on the protein expression of (A) The protein band detection results, (B) phospho-NF-κB, and C. IκB-α. Cells were preincubated for 24 h and then treated with 1 μg/ml LPS and BK-5 exosomes (1.69 × 10^7^, 3.38 × 10^7^, 6.75 × 10^7^ particles/ml). Phosphorylation of NF-κB and IκB-α was analyzed by western blotting. Data represent the means ± standard deviation with three independent experiments. **p < 0.01; ***p < 0.001. BK-5 exosomes, *Latilactobacillus sakei*–derived exosomes; LPS, lipopolysaccharide; NF-κB, nuclear factor kappa-light-chain-enhancer of activated B cells; IκB-α, inhibitor of κB alpha.

The NF-κB signaling pathway plays a central role in mediating inflammatory responses. Upon LPS stimulation, IκB-α is degraded, enabling phosphorylated NF-κB (p-NF-κB) to translocate into the nucleus and induce the transcription of proinflammatory genes [44]. Therefore, we assessed the changes in p-NF-κB and IκB-α levels to evaluate the regulatory effects of BK-5 exosomes on inflammation. Western blot analysis showed a concentration-dependent decrease in p-NF-κB expression, accompanied by an increase in IκB-α levels (Fig. 11).

### Inhibition of TRP-1, TRP-2, Tyrosinase, and MITF Protein Expression

**Fig. 12:**
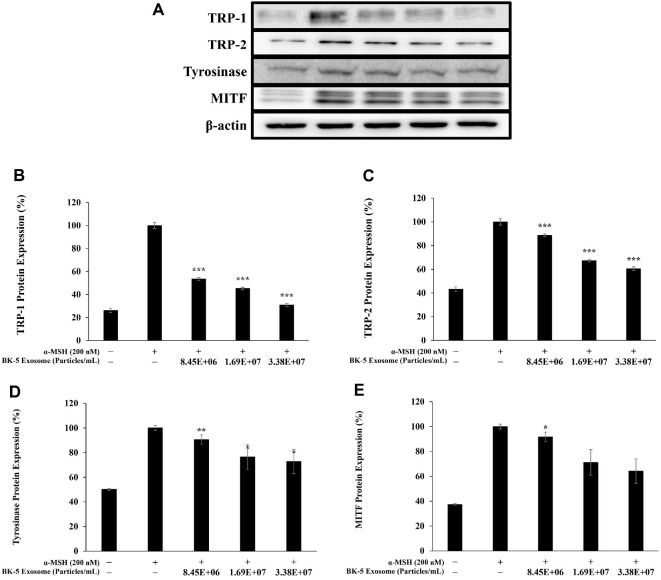
Western blot analysis of tyrosinase-related protein (TRP)-1, TRP-2, tyrosinase, and microphthalmiaassociated transcription factor (MITF) in α-MSH-induced B16F10 melanoma cells. Cells were pre-incubated for 48 h and then treated with α-MSH (200 nM) and BK-5 exosomes (8.45 × 10^6^, 1.69 × 10^7^, and 3.38 × 10^7^ particles/ml). (**A**) The protein band detection results, (**B**) TRP-1, (**C**)TRP-2, (**D**) tyrosinase, and (**E**) MITF protein levels were analyzed using western blotting. β-actin was used as the control. Data represent the means ± standard deviation with three independent experiments. *p < 0.05; **p < 0.01; ***p < 0.001. BK-5 exosomes, *Latilactobacillus sakei*–derived exosomes; α-MSH, alpha-melanocytestimulating hormone.

MITF regulates melanin synthesis by controlling the expression of its downstream target genes, including tyrosinase, TRP1, and TRP2 [45]. Tyrosinase catalyzes the oxidation of L-tyrosine into L-DOPA and DOPA-quinone, initiating melanin biosynthesis, while TRP-1 and TRP-2 function as key enzymes involved in the subsequent stabilization and regulation of melanin synthesis. Therefore, to evaluate the effects of BK-5 exosomes on melanogenesis, protein levels of MITF and its downstream targets—tyrosinase, TRP-1, and TRP-2—were assessed. Western blot analysis revealed a concentration-dependent reduction in the expression of TRP-1, TRP-2, tyrosinase, and MITF (Fig. 12).

## Discussion

In this study, we verified the anti-inflammatory and skin-lightening activities of BK-5 exosomes. The exosomes were purified using a TFF system, and their characteristic size and morphology were confirmed using NTA and TEM [46]. Our findings demonstrated consistent antioxidant activity of BK-5 exosomes across all tested concentrations, with quantitative analysis expressed on a per-particle basis. At the highest concentration (6.75 × 10^7^ particles/ml), the radical scavenging activity exceeded 85%, demonstrating strong antioxidant potential [47]. These antioxidant properties may also be associated with the potential anti-inflammatory and skin-lightening effects of the exosomes, as they could contribute to alleviating oxidative stress during inflammatory responses.

Treatment of RAW 264.7 macrophages with BK-5 exosomes at non-cytotoxic concentrations (1.69 × 10^7^, 3.38 × 10^7^, and 6.75 × 10^7^ particles/ml) significantly reduced NO production in a concentration-dependent manner to 24.2%, 44.8%, and 59.8%, respectively. Previously, exosomes derived from LAB have been shown to significantly suppress NO production in RAW 264.7 macrophages, and this observation is consistent with reports that exosomes originating from LAB species such as *Leuconostoc* similarly inhibit NO generation [48]. The concomitant suppression of IL-6 and IL-1β secretion was mechanistically associated with decreased NO and PGE_2_ levels, indicating that BK-5 exosomes exert multifaceted anti-inflammatory effects by modulating several inflammatory mediators. In contrast, TNF-α inhibition was relatively modest, possibly due to differences in activation kinetics or pathway specificity, suggesting the need for time-course analyses in future investigations. TNF-α, IL-1β, and IL-6 are among the commonly used pro-inflammatory markers in LPS-activated RAW 264.7 macrophages, and they have been reported to function as direct downstream targets of the NF-κB and p38 MAPK signaling pathways [49]. In addition, many previous inflammation studies have employed the same cytokine panel. Therefore, these three factors were selected in the present study to ensure comparability of results and consistency in data interpretation.

Mechanistic analyses revealed that BK-5 exosomes moderately inhibited ERK phosphorylation but strongly suppressed p38 MAPK phosphorylation. The pronounced inhibition of p38 may be associated with the biological feature that p38 more directly regulates the expression of inflammatory genes such as iNOS, COX-2, IL-6, and IL-1β compared with ERK and JNK [50]. This differential response may be attributable to variations in exosomal cargo composition or strain-specific characteristics. while preventing IκB-α degradation and nuclear translocation of NF-κB. These findings indicate that BK-5 exosomes attenuate inflammatory signaling, primarily through inhibition of the p38–NF-κB axis, leading to decreased expression of iNOS and COX-2 and consequent downregulation of proinflammatory gene expression.

The skin-lightening potential of BK-5 exosomes was evaluated in B16F10 melanoma cells treated at non-cytotoxic concentrations (8.45 × 10^6^, 1.69 × 10^7^, and 3.38 × 10^7^ particles/ml). Melanin content was reduced by 33.2%, 39.5%, and 50.4%, respectively, accompanied by decreases in tyrosinase activity by 18.4%, 28.7%, and 47.9%, respectively. Western blot analysis further demonstrated that BK-5 exosomes downregulated MITF and its downstream targets, TRP-1, TRP-2, and tyrosinase. This level of inhibition is more pronounced than the typical range of melanin reduction reported for natural or microbial-derived materials [51]. These findings indicate that BK-5 exosomes suppress the overall melanogenic pathway not merely through tyrosinase inhibition but by regulating MITF-mediated transcription. Such mechanistic characteristics provide molecular evidence supporting the potential application of BK-5 exosomes as a functional whitening agent.

Collectively, BK-5 exosomes derived from the flower-associated *L. sakei* exhibited dual functionality-anti-inflammatory and skin-lightening activities-highlighting their potential as multifunctional bioactive agents. These findings suggest that BK-5 exosomes may serve as promising candidates for alleviating skin inflammation and pigmentation, with applications in both cosmetic and therapeutic contexts. The multifactorial mechanism of exosome-based regulation offers an advantage over conventional single-compound approaches. Future studies should aim to elucidate molecular mechanisms at the transcriptomic and proteomic levels, identify bioactive factors, validate efficacy in *in vivo* models, and assess clinical applicability in human systems.

## Figures and Tables

**Fig. 9 F9:**
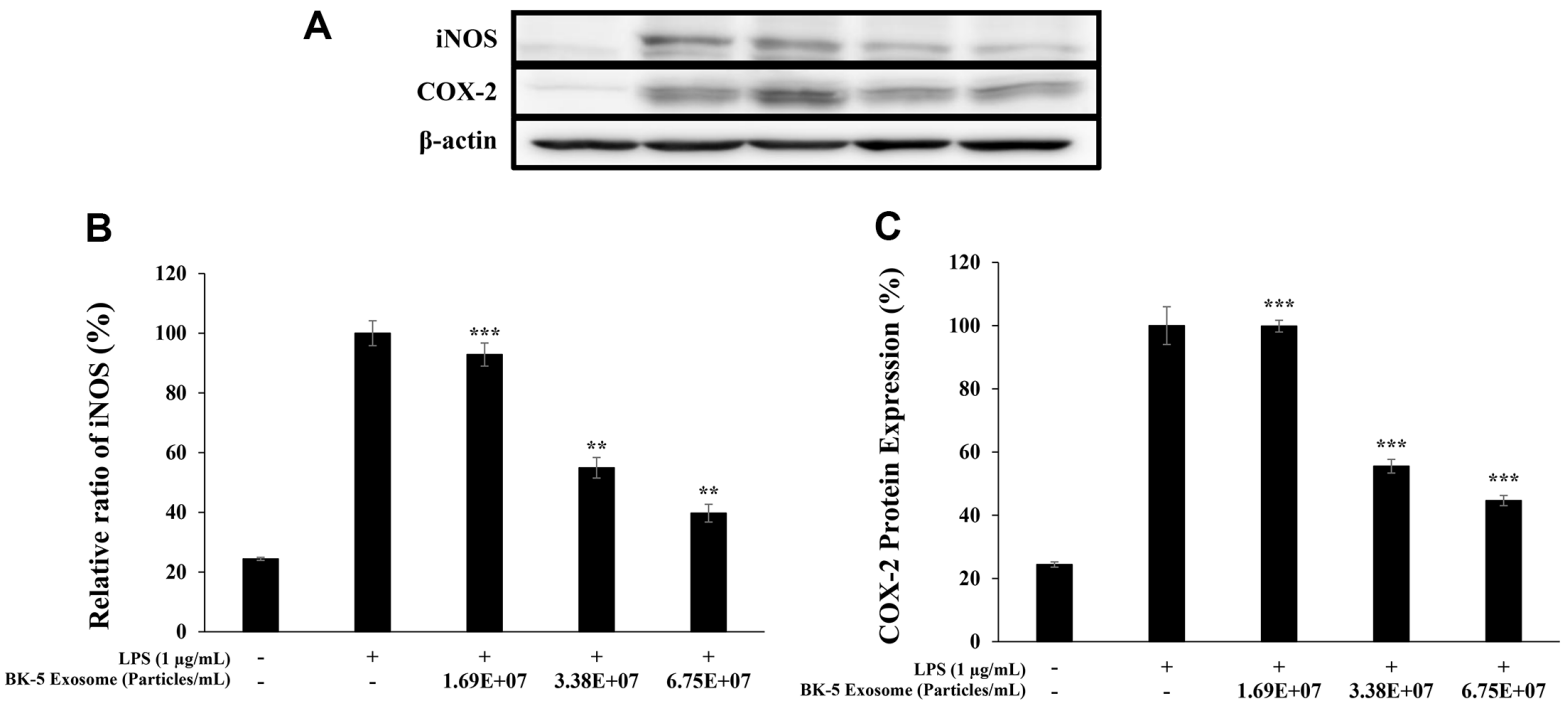
Inhibitory effects of BK-5 exosomes on inducible nitric oxide synthase (iNOS) and cyclooxygenase 2 (COX-2) protein expression in LPS-stimulated RAW 264.7 cells. Inhibitory effect of BK-5 exosome on the protein levels of (**A**) The protein band detection results, (**B**) iNOS, and (**C**) COX-2 in RAW 264.7 cells stimulated with LPS (1 μg/ml) in the presence of BK-5 exosomes (1.69 × 10^7^, 3.38 × 10^7^, and 6.75 × 10^7^ particles/ml). Expression of iNOS, COX-2, and β-actin was determined by western blotting. Data represent the means ± standard deviation with three independent experiments. **p < 0.01; ***p < 0.001. BK-5 exosomes, *Latilactobacillus sakei*–derived exosomes; LPS, lipopolysaccharide.
